# Effects of two kinds of vestibular function training on reducing motion sickness in college students

**DOI:** 10.3389/fneur.2025.1433065

**Published:** 2025-02-06

**Authors:** Linyao Shi, Jing Zhao, Jiamei Lu, Chuanxia Cao, Qikun Zhang, Chuanjing Qiu, Zhanguo Jin, Shengguang Yan

**Affiliations:** ^1^School of Public Health, North China University of Science and Technology, Tangshan, China; ^2^Research Center for Air and Space Medicine and Vertigo Diagnosis and Treatment, Air Force General Hospital PLA, Beijing, China; ^3^Hebei Key Laboratory of Occupational Health and Safety for Coal Industry, Tangshan, China; ^4^Hebei Coordinated Innovation Center of Occupational Health and Safety, Tangshan, China

**Keywords:** motion sickness, Graybiel, electric rotating chair, heart rate variability, visual-motion cage rotating chair

## Abstract

**Introduction:**

To explore the advantages and disadvantages of different vestibular function training to improve Motion sickness (MS) can be associated with significant symptoms, including fatigue, dizziness, headaches, nausea, and vomiting. Vestibular function training has increasingly replaced MS medications over the past few years and has almost no side effects.

**Methods:**

We selected 109 students with MS from a university in Tangshan, China, and randomly assigned them to either an electric rotating chair group or a visual-motion cage rotating chair group. Both training groups underwent vestibular function training for 90 seconds a day for seven consecutive days.

**Results:**

After training, both groups’ Graybiel scores, blood pressure, high-frequency power (HF), and root mean square of successive differences (rMSSD) between adjacent normal heartbeats significantly decreased. In the visual-motion cage rotating chair group, in addition to a reduction in the percentage of adjacent normal-to-normal intervals which differed by more than 50 ms (pNN50), as well as decreases in low-frequency power (LF), an increase in LF/HF was observed. Between-group comparisons showed that the Graybiel scores in the electric rotating chair group were better than those in the visual-motion cage rotating chair group. When the two groups were stratified into high and low-susceptibility subgroups, the low-susceptibility subgroup of the electric rotating chair group had lower Graybiel scores and diastolic blood pressures than the low-susceptibility visual-motion cage rotating chair subgroup, whereas in the high susceptibility subgroup, LF, rMSSD, and pNN50 were significantly higher in the visual-motion cage rotating chair group than in the electric rotating chair group.

**Discussion:**

This study compared the effects of two types of vestibular function training on Graybiel scores and heart rate variability (HRV). we found that seven days of training with both the electric rotating chair and the visual-motion cage rotating chair relieved MS symptoms and enhanced sympathetic regulation. In terms of improving the subjective degree of MS, the electric rotating chair was superior to the visual-motion cage rotating chair. Subgroup analysis results showed that low-susceptibility MS patients’ symptoms were better relieved with electric rotating chair training, while high-susceptibility patients’ symptoms were better relieved with visual-motion cage rotating chair training.

## 1 Introduction

Motion Sickness (MS) is caused by a conflict between the body’s somatic and visual sensations, visceral sensors, and vestibular nerve inputs. It can be related to real or illusory motion and leads to symptoms including nausea and vomiting (1). In the general population, 5% of people experience severe car sickness, 5% are unaffected, and the rest experience moderate car sickness (2). MS reduces cognitive functioning and affects the sensor experiences of space and balance. In some security-sensitive activities that require accurate performance and spatial positioning, MS will not only limit the combat effectiveness of naval forces, affect their work efficiency, but also cause personal safety problems in severe cases (3, 4).

The pathogenesis of MS has not been fully clarified, but the neural mismatch theory is the most widely accepted explanation (5). This theory suggests that the central nervous system memorizes past motion stimuli, and postulates that MS is induced with an actual motion-related situation that does not match the stored neural signals. Many scholars have proposed different treatment methods for MS, including medications, traditional Chinese medicine (TCM), and vestibular function training (6, 7). Most MS medications can cause adverse reactions such as blurry vision, dry mouth, and nausea (8), all of which affect quality of life. TCM treatments tend to have slow onsets of action (9), and there are relatively few trained TCM practitioners. Vestibular function training, however, has almost no side effects and only requires healthcare workers to operate the equipment, making it a safe and effective treatment option (10).

Evaluating the effects of vestibular function training on MS symptoms requires objective indicators. The most commonly used evaluation metrics include the MS Susceptibility Questionnaire (MSSQ) (11), Graybiel’s MS assessment questionnaire (12), blood pressure (BP) (13), heart rate (HR) (14), and heart rate variability (HRV) (15, 16). HRV quantitatively reflects autonomic nervous system activity, and can be used to examine the regulatory strength of the sympathetic and vagus nerves, monitor training effects, and prevent overtraining injuries (17, 18). Wang et al. (19) conducted a maritime trial and found that subjects’ high-frequency power (HF) increased and low-frequency/high-frequency power ratios (LF/HF) decreased during the voyage but returned to normal after landing. Pan et al. (20) found that both and diastolic BP were significantly increased after rotating chair stimulation, with no significant changes in HR. These results contradict Wang et al.’s findings (4), which showed no significant changes in BP but did show HR accelerations after electric rotating chair stimulation. Holmes et al. (21) found that the severity of HRV changes in response to visual-vestibular rotation stimulation was related to MS susceptibility. Thus, past research suggests that these indicators can all be used to evaluate the effectiveness of vestibular function training.

Although there are numerous vestibular functional training methods, most involve passive training. Passive training involves the body passively receiving training from devices such as electric rotating chairs and visual-motion cage rotating chairs (22) Huang et al. (23) conducted a seven-day electric rotating chair training and found that it extended participants’vestibular stimulation tolerance times and improved vestibular system stability. Ressiot et al. (24) conducted a visual-motion cage rotating chair training and found that it enhanced vestibular function stability and alleviated MS symptoms. However, due to differences in training methods and/or training environments, it is often difficult to compare the effects of different vestibular function training. Additionally, most studies only evaluate singular passive training effects, with few comparing the effects of two or more passive trainings, and even fewer examining the efficacy of electric rotating chair and visual-motion cage rotating chair training for participants with different MS susceptibilities.

Thus, the purpose of this study was to compare the efficacy of electric rotating chairs and visual-motion cage rotating chairs in improving subjective MS symptoms and sympathetic vascular regulatory capacity. We also aimed to explore suitable training programs for patients with different MS susceptibilities. We predicted that both electric rotating chairs and visual-motion cage rotating chairs could treat MS, but that the effects of electric rotating chairs would be superior to those of visual-motion cage rotating chairs.

## 2 Materials and methods

### 2.1 Design of experiment

This study was a completely randomized design. 109 participants with MS were recruited through random selection from a university in Tangshan, China. All students had previously completed the MSSQ-S questionnaire. MSSQ-S is a short form of the Motion Sickness Susceptibility Questionnaire used to assess individuals’ sensitivity to motion sickness. Typically, individuals are only required to answer the questionnaire questions based on their experiences. The questionnaire includes questions about experiencing nausea and vomiting when using transportation or engaging in entertainment during childhood (before 12 years old) and adulthood. The measurement of MSSQ-S was conducted through an online questionnaire. Participants were randomly assigned to either an electric rotating chair group (case number = 55) or a visual-motion cage rotating chair group (case number = 54). The MSSQ-S ranged from 1 to 53 in both the electric rotating chair group and the visual-motion cage rotating chair group.

### 2.2 Experimental subjects

We included participants who were ≥ 18 years old, had a Graybiel questionnaire score ≥ 1 point after electric rotating chair stimulation, had no vestibular dysfunction, gave verbal confirmation of not having used any central nervous system-affecting drugs in the week before the trial, and did not consume any alcohol or coffee in the 24 h before the experiment. This is to control as much as possible the factors that affect the results of the experiment. Individuals with inner ear disease, hypertension, diabetes, heart disease, or other serious physical conditions, as well as those with evident anxiety and depression, claustrophobia, inability to cooperate, or a history of treatment interruption or noncompliance, were excluded. This study was approved by the Air Force Medical Center of the People’s Liberation Army of China Ethics Committee (2022-203-PJ01). All participants signed an informed consent form. The study was conducted by relevant guidelines and legislation.

### 2.3 Experimental protocol

It was verbally confirmed that the subjects had not taken any medication that affected central nervous system function in the week before the test and had not consumed alcohol or coffee within 24 h before the test. This study was a quasi-randomized controlled trial. The subjective and objective indicators of the same training group after training and before training were experimental and control, and there was no blank control group.

On the zero-day, both participant groups were stimulated using an electric rotating chair (Beijing Hangsu Technology Co., Ltd., HSZY-1 model) to induce MS symptoms. The stimulation protocol was as follows: The chair was set to rotate clockwise at 180°/s. Subjects were instructed to close their eyes and, following the metronome’s cues, sway their heads left and right against the headrest, with a swaying angle of 30°to each side and a frequency of once every two seconds, and for a duration of 90 s.

For the first to the seventh day, participants underwent training using either the electric rotating chair (Beijing Hangsu Technology Co., Ltd., HSZY-1 model) or the visual-motion cage rotating chair (Beijing Hangsu Technology Co., Ltd., HS-SLD-TYKT model). The electric rotating chair training protocol was consistent with the stimulation protocol, with one session per day. For the visual-motion cage rotating chair training, the chair was set to rotate clockwise at 180°/s. Participants were instructed to keep their eyes open and fixate on the visual-motion drum (decorated with a black and white chessboard pattern), which rotated counterclockwise at 180°/s, for a training duration of 90 s, for one session per day.

### 2.4 Indicators of evaluation

#### 2.4.1 Subjective indicators

This study used the Graybiel MS assessment questionnaire 28 (Supplementary Table S1) to evaluate the participants’ subjective feelings. All participants completed it immediately after each day’s training. Graybiel scores were calculated based on questionnaire contents. If a participant requested to stop due to intolerance, a Graybiel corrected score was calculated based on the actual rotation time (Corrected Score = Actual Score ×90 / Actual Rotation Seconds). According to the Graybiel score of the subjects on day 1 after stimulation, we divided them into low-high-susceptibility subjects with a median score of 7 [1–7 as low-susceptibility subjects, >7 as high-susceptibility subjects (25)]. In this study, the Graybiel score questionnaire filled out after the induction experiment was used as the baseline data, and the measured data after the training were compared. If the Graybiel score decreased after the training, the training effect was effective.

#### 2.4.2 Blood pressure

A user-friendly and rapid detection blood pressure monitor (Tianjin Jiuan Electronic Co., Ltd., iHealth BP5 model) was used to measure the participants’ systolic blood pressure, diastolic blood pressure, and heart rate before and after each day’s training. Participants were instructed to sit quietly for five minutes before the pre-training measurement, and the post-training measurement was taken immediately afterward. In this study, the blood pressure measured after the induction experiment was used as the baseline data to compare with the data measured after training. If the systolic blood pressure and diastolic blood pressure were significantly reduced after training, the training effect was effective.

#### 2.4.3 Heart rate variability and heart rate

A convenient-to-wear and accurate HRV measuring device (Nanjing FSYK Software Technology Co., Ltd., AECG-600D model) was used to measure the participants’ high-frequency power (HF), low-frequency power (LF), LF/HF ratio, root mean square of successive differences (rMSSD), and the percentage of adjacent normal-to-normal intervals which differed by more than 50 ms (pNN50). Measurements were taken on the first day before and after training and after training on the eighth day. Subjects were instructed to sit quietly for five minutes before each measurement. In this study, the heart rate variability and heart rate measured after the induction experiment were used as the baseline data to compare with the data measured after training. If HF, LF, rMSSD, pNN50, and heart rate decreased after training, and LF/HF increased, the training effect was effective.

### 2.5 Sample size calculation

Based on the literature (26), the average LF parameter for low MS susceptibility groups is 978.19 ± 645.21 ms^2^/Hz, and the average LF parameter for high MS susceptibility groups is 1411.31 ± 634.19 ms^2^/Hz. From this, S = 634.19, and *δ* = 1411.31–978.19 = 433.12. With a test level *α* of 0.05 and *β* set to 0.20, each group needed 23 participants (based on the formula for the required sample size in A/B testing). Including an assumed 10% loss to follow-up, we calculated the minimum total sample size for this study to be 102 participants.


$$n=\frac{S^{2}}{\delta^{2}}{\left(Z_{\alpha /2}\pm Z_{\beta }\right)}^{2}$$

### 2.6 Randomization

#### 2.6.1 Sequence generation

The subjects were numbered according to the time sequence of enrollment, and each number corresponded to the student number of one research subject and filled in the Excel table accordingly.

#### 2.6.2 Implement randomization

Generate a random number table in the Excel table and assign random numbers to each subject; the random numbers were sorted according to the rule from largest to smallest. The first half of the subjects are specified as the same group, that is, the electric rotating chair group; the latter half of the subjects were the same group, that is, the Visual-motion cage rotating chair group.

#### 2.6.3 Blind method

Blinding was not used because the processing factors of the two groups could be perceived by the researchers and subjects during the experiment.

### 2.7 Statistical analysis

An Excel database was established, and data were analyzed using SPSS (version 23.0). Quantitative data that followed a normal distribution are presented as $\bar{X}$ *± S*, while data that did not follow a normal distribution are presented as *M* (*P*_25_, *P*_75_). Paired samples *t*-tests or Wilcoxon tests were used for within-group comparisons; independent-samples *t*-tests, Satterthwaite *t*-tests, or Kolmogorov–Smirnov tests were used for between-group comparisons. We used paired samples *t*-tests for within-group data that followed normal distributions, and Wilcoxon tests for within-group data that did not follow normal distributions. For between-group comparisons, we used independent-samples *t*-tests for data that were normally distributed and had variance homogeneity, Satterthwaite *t*-tests for normally distributed data without homogeneity of variance, and Kolmogorov–Smirnov *Z* tests for data that met neither assumption. Categorical data are presented as rates or proportions, with comparisons between groups completed with Chi-square tests. The test level *α* was set at 0.05, and all tests were two-sided.

## 3 Results

### 3.1 Comparison of baseline levels between the two groups of subjects

A comparison of baseline levels between the two groups of subjects was conducted, which showed no statistical differences in the indicators before training, indicating comparability between the groups (*p* > 0.05) (Table 1).

**Table 1:** Comparison of baseline levels between the two groups of subjects.

Variable	Electric rotating chair group (*n* = 55)	Visual-motion cage rotating chair group (*n* = 54)	*t/Z/χ^2^*	*P*
Age, year	19.00 (19.00, 21.00)	20.00 (19.00, 21.00)	0.895^c^	0.400
Case, m/f	55.0 (16.0/39.0)	54.0 (15.0/39.0)	0.023^a^	0.879
MSSQ-S, points	36.21 (6.00, 42.90)	20.15 (6.29, 38.55)	1.313^c^	0.064
Systolic pressure, mmHg	131.00 (119.00, 138.00)	123.00 (114.75, 135.00)	1.018^c^	0.252
Diastolic pressure, mmHg	83.27 ± 12.43	79.57 ± 9.30	1.757^b^	0.082
Heart rate, rpm	79.00 (73.00, 88.00)	80.00 (73.50, 87.00)	0.566^c^	0.906
LF, ms^2^/Hz	1225.22 (921.89, 1617.50)	1384.80 (863.59, 1805.86)	0.995^c^	0.276
HF, ms^2^/Hz	702.35 (390.44, 1131.15)	668.95 (450.94, 1058.83)	0.634^c^	0.816
LF/HF	1.83 (1.33, 2.65)	2.20 (1.53, 2.72)	1.188^c^	0.119
rMSSD, ms	38.01 (27.82, 49.91)	39.02 (30.89, 44.38)	0.842^c^	0.478
pNN50, %	16.35 (7.72, 28.45)	18.06 (10.58, 25.72)	0.838^c^	0.483
Graybiel, points	7.00 (4.00, 13.00)	7.50 (2.75, 16.50)	0.599^c^	0.865

MSSQ-S: motion sickness susceptibility questionnaire-short score, LF: low-frequency component, HF: high-frequency component, Hz: Hertz, rMSSD: root mean square of the successive differences, pNN50: Percentage of adjacent normal-to-normal intervals differing by more than 50 ms. ^a^Chi-square tests; ^b^two independent sample *t*-tests; ^c^Kolmogorov–Smirnov *Z* tests; ^d^Proportion of subjects with low/high susceptibility in electric and visual-motion cage rotating chair.

### 3.2 Subject and objective index analysis of two groups before and after training

**Figure 1:**
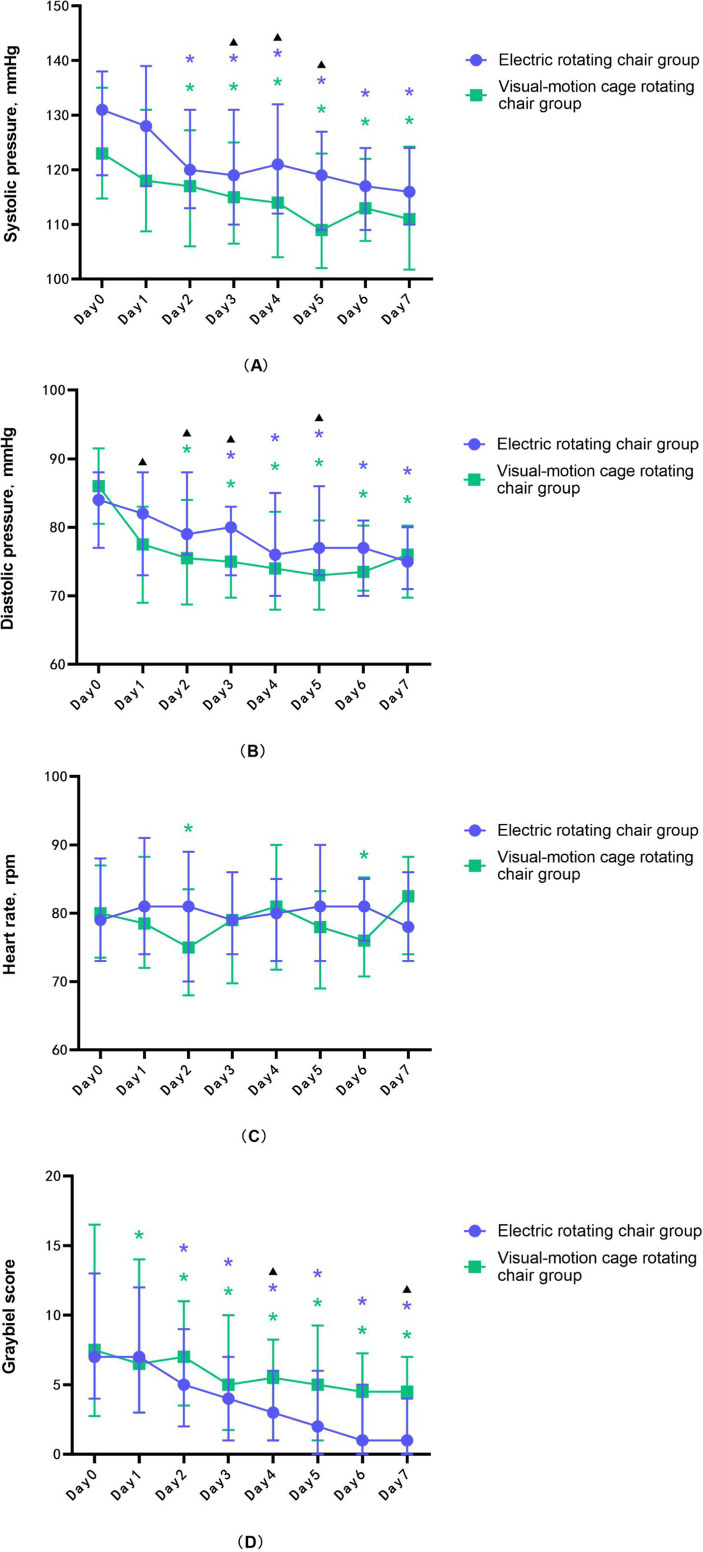
The changing trend of **(A)** systolic blood pressure, **(B)** diastolic blood pressure, **(C)** heart rate, and **(D)** Graybiel score during the training of the electric rotating chair group and the visual-motion cage rotating chair group. Error lines indicate median and quartile, purple * represents a significant difference in the electric rotating chair group, compared to day 0, green * represents a significant difference in the visual-motion cage rotating chair group, compared to day 0, and black ▴represents significant differences between the two training groups.

The results for the electric rotating chair group demonstrated that, compared to before training, the subjects’ Graybiel scores (Figure 1D), systolic blood pressure (Figure 1A), and diastolic blood pressure (Figure 1B) were significantly decreased (*p* < 0.001), while heart rate (Figure 1C) was not statistically significant before and after training (*p* > 0.05). Specifically, the Graybiel score decreased by five points, suggesting a significant reduction in MS symptoms. Systolic blood pressure decreased by 16.0 mmHg, diastolic blood pressure decreased by 7.9 mmHg, and HF and rMSSD also significantly decreased (*p* < 0.05), indicating a reduction in sympathetic nervous system excitability in subjects trained with the electric rotating chair (Table 2).

**Table 2:** Effect evaluation before and after vestibular function training in the electric rotating chair group.

Variable	Pre-training	Post-training	Difference value	*t/Z*	*P*
Graybiel, points	7.0 (4.0, 13.0)	1.0 (0.0, 4.0)	5.0 (3.0, 10.0)	-6.047^b^	<0.001
Systolic pressure, mmHg	131.0 (119.0, 138.0)	116.0 (110.0, 124.0)	16.0 (4.0, 23.0)	−5.323^b^	<0.001
Diastolic pressure, mmHg	83.3 ± 12.4	75.4 ± 9.4	7.9 ± 13.6	4.299^a^	<0.001
Heart rate, rpm	79.0 (73.0, 88.0)	78.0 (73.0, 86.0)	3.0 (−10.0, 11.0)	−0.589^b^	0.556
LF, (ms^2^/Hz)	1225.2 (921.9, 1617.5)	1020.4 (687.4, 1488.1)	24.2 (−237.0, 523.6)	1.356^b^	0.181
HF, (ms^2^/Hz)	702.4 (390.4, 1131.2)	502.2 (269.0, 984.6)	188.7 (−78.8, 421.1)	−2.840^b^	0.005
LF/HF	1.8 (1.3, 2.7)	2.0 (1.4, 3.2)	−0.1 (−1.0, 0.2)	−1.908^b^	0.056
rMSSD, ms	38.0 (27.8, 49.9)	33.0 (25.7, 48.1)	6.3 (−5.8, 10.1)	−2.007^b^	0.045
pNN50, %	16.4 (7.7, 28.5)	12.2 (5.7, 25.9)	3.7 (−4.7, 8.0)	−1.734^b^	0.083

a Paired samples *t*-tests.

b Wilcoxon tests.

The results for the visual-motion cage rotating chair group demonstrated that, compared to before training, the subjects’ Graybiel scores (Figure 1D), systolic blood pressure (Figure 1A), diastolic blood pressure (Figure 1B), rMSSD, pNN50, and HF were also significantly decreased (*p* < 0.001), while heart rate (Figure 1C) was not statistically significant before and after training (*p* > 0.05). In this group, average Graybiel scores decreased by three points; systolic blood pressure decreased by 9.9 mmHg; diastolic blood pressure decreased by 4.0 mmHg; and HRV indicators including HF, rMSSD pNN50, and LF all decreased, although LF/HF increased (Table 3). These findings indicate that visual-motion cage rotating chair training also improved subjective tolerance to MS and HRV stability.

**Table 3:** Effect evaluation of vestibular function training before and after visual-motion cage rotating chair group.

Variable	Pre-training	Post-training	Difference value	*t/Z*	*P*
Graybiel, points	7.5 (2.8, 16.5)	4.5 (0.0, 7.0)	3.0 (1.0, 10.0)	−4.681^b^	<0.001
Systolic pressure, mmHg	122.8 ± 14.5	112.9 ± 13.3	9.9 ± 10.3	7.062^a^	<0.001
Diastolic pressure, mmHg	80.5 (73.0, 86.0)	76.0 (69.8, 80.3)	4.0 (0.0, 9.3)	−3.604^b^	<0.001
Heart rate, rpm	80.4 ± 9.6	81.6 ± 9.5	−1.3 ± 9.8	−0.946^a^	0.348
LF, ms^2^/Hz	1384.8 (863.6, 1805.9)	1069.4 (703.0, 1384.9)	177.0 (−163.4, 662.6)	−2.932^b^	0.003
HF, ms^2^/Hz	668.9 (450.9, 1058.8)	459.4 (287.6, 754.0)	166.0 (2.9, 524.2)	−3.741^b^	<0.001
LF/HF	2.2 (1.5, 2.7)	2.3 (1.7, 3.5)	−0.2 (−1.0, 0.5)	−2.342^b^	0.019
rMSSD, ms	39.0 (30.9, 44.4)	33.2 (23.9, 41.1)	3.6 (−1.1, 11.4)	−3.621^b^	<0.001
pNN50, %	18.1 (10.6, 25.7)	13.3 (4.5, 20.9)	4.8 (−0.5, 10.1)	−3.466^b^	<0.001

a Paired samples *t*-tests.

b Wilcoxon tests.

### 3.3 Subjective and objective index analysis between two training groups

The comparison results of the two groups showed that the only statistically significant difference between the two groups was in Graybiel scores (Table 4) (Figure 1D), which suggested that the electric rotating chair was superior to the visual-motion cage rotating chair in alleviating subjective perceptions of motion sickness.

**Table 4:** Comparison of the difference values of each index before and after the two vestibular function training.

Variable	Electric rotating chair group (*n* = 55)	Visual-motion cage rotating chair group (*n* = 54)	*t/Z*	*P*
Graybiel, points	5.0 (3.0, 10.0)	3.0 (1.0, 10.0)	1.367^b^	0.048
Systolic pressure, mmHg	16.0 (4.0, 23.0)	11.0 (−0.3, 18.0)	1.128^b^	0.157
Diastolic pressure, mmHg	8.0 (2.0, 18.0)	4.0 (0.0, 9.3)	1.132^b^	0.154
Heart rate, rpm	0.9 ± 14.6	−1.3 ± 9.8	0.897^a^	0.372
LF, ms^2^/Hz	24.2 (−237.0, 523.6)	177.0 (−163.4, 662.6)	0.824^b^	0.505
HF, ms^2^/Hz	188.7 (−78.8, 421.1)	166.0 (2.9, 524.2)	0.643^b^	0.802
LF/HF	−0.1 (−1.0, 0.2)	−0.2 (−1.0, 0.5)	0.703^b^	0.706
rMSSD, ms	6.3 (−5.8, 10.1)	3.6 (−1.1, 11.4)	1.035^b^	0.234
pNN50, %	3.7 (−4.7, 8.0)	4.8 (−0.5, 10.1)	0.933^b^	0.348

a Satterthwaite *t*-test.

b Kolmogorov–Smirnov *Z* test.

### 3.4 The effect of acclimatization training was different between the two groups with low and high susceptibility

There were no statistical differences in the baseline levels of indicators between the low and high susceptibility subgroups, indicating comparability (*p* > 0.05) (Supplementary Table S2).

**Figure 2:**
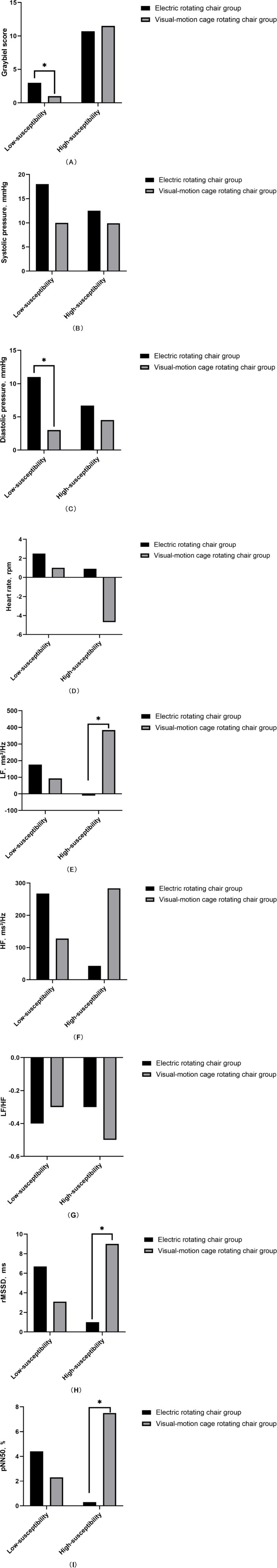
Comparison of subjective and objective indicators between the two training groups for low and high susceptibility subjects. *p < 0.05.

The results indicated that there were statistically significant differences in Graybiel score (Figure 2A) and diastolic blood pressure (Figure 2C) among low-susceptibility subjects, while other indicators showed no statistically significant differences (Figure 2). Furthermore, the reduction of Graybiel score and diastolic blood pressure in low-susceptibility subjects was found to be more effective than that in the visual-motion cage rotating chair group (*p* < 0.05) (Table 5).

**Table 5:** Difference of acclimation training effect among low-susceptibility subjects.

Variable	Electric rotating chair group (*n* = 28)	Visual-motion cage rotating chair group (*n* = 27)	*t/Z*	*P*
Graybiel, points	3.0 (1.3, 5.0)	1.0 (0.0, 3.0)	1.545^b^	0.017
Systolic pressure, mmHg	18.0 (5.3, 23.8)	10.0 (0.0, 18.0)	1.045^b^	0.225
Diastolic pressure, mmHg	11.0 (5.0, 20.8)	3.0 (0.0, 10.0)	1.555^b^	0.016
Heart rate, rpm	2.5 (−10.0, 11.0)	1.0 (−3.0, 5.0)	1.192^b^	0.117
LF, ms^2^/Hz	176.1 (−242.2, 771.1)	92.9 (−247.0, 418.0)	0.902^b^	0.389
HF, ms^2^/Hz	267.5 (−0.8, 546.5)	127.9 (−34.8, 283.1)	1.035^b^	0.235
LF/HF	−0.4 ± 1.3	−0.3 ± 1.2	−0.134^a^	0.894
rMSSD, ms	6.7 (−4.9, 15.2)	3.1 (−1.6, 6.8)	1.054^b^	0.261
pNN50, %	4.4 (−3.0, 12.0)	2.3 (−1.6, 7.4)	1.040^b^	0.230

a Two independent sample *t*-tests.

b Kolmogorov–Smirnov *Z* test.

The results indicated that LF (Figure 2E), rMSSD (Figure 2H), and pNN50 (Figure 2I) exhibited statistically significant differences in the high-susceptibility group, while other indicators showed no statistically significant differences (Figure 2). The reduction of LF, rMSSD, and pNN50 in the high susceptibility group showed superior efficacy compared to the electric rotating chair group (*p* < 0.05) (Table 6).

**Table 6:** Difference of acclimatization training effect among high-susceptibility subjects.

Variable	Electric rotating chair group (*n* = 27)	Visual-motion cage rotating chair group (*n* = 27)	*t/Z*	*P*
Graybiel, score	10.7 ± 6.4	11.5 ± 9.9	−0.337^a^	0.738
Systolic pressure, mmHg	12.5 ± 12.1	9.9 ± 9.8	0.866^a^	0.390
Diastolic pressure, mmHg	6.7 ± 11.7	4.5 ± 7.7	0.838^a^	0.406
Heart rate, rpm	0.9 ± 12.3	−4.7 ± 9.0	1.890^a^	0.064
LF, ms^2^/Hz	−10.8 (−234.7, 333.1)	383.2 (9.6, 816.3)	1.361^b^	0.049
HF, ms^2^/Hz	43.1 (−184.6, 311.4)	283.9 (45.7, 553.3)	1.089^b^	0.187
LF/HF	−0.3 ± 1.1	−0.5 ± 1.3	0.769^a^	0.445
rMSSD, ms	1.0 ± 9.9	9.0 ± 13.5	−2.477^a^	0.017
pNN50, %	0.3 ± 9.1	7.5 ± 12.6	−2.428^a^	0.019

a Two independent sample *t*-tests.

b Kolmogorov–Smirnov *Z* test.

## 4 Discussion

This study offers new insights into the treatment of MS. We demonstrated that both electric rotating chairs and visual-motion cage rotating chairs could treat MS. Additionally, the electric rotating chair was more effective than the visual-motion cage rotating chair, supporting our hypothesis. The electric rotating chair relieved numerous symptoms of MS, including dizziness, vomiting, and nausea, and was found to be more suitable for individuals with low MS susceptibility.

### 4.1 The vestibular system regulates the autonomic response

There are many mechanisms for vestibular function training to improve motion sickness. A recent study showed that cholecystokinin-expressing (CCK) vestibular nucleus (VN) neurons elicit autonomic responses in MS after activation, suggesting that vestibular function training may alleviate subsequent motion sickness symptoms by stimulating vestibular organs to a certain extent, leading to changes in the central vestibular nuclei and CCK-expressing VN neurons, which in turn alleviates subsequent motion sickness symptoms (27). Another study showed that (28), the vestibular system regulates cardiovascular functions in two specific ways: (1) It perceives changes in linear acceleration and generates neural signals, which are transmitted to the autonomic nervous system, which then regulates sympathetic and vagus activity based on signal intensity, leading to changes in BP and HRV (29) (2) Neural signals are transmitted to the hypothalamic–pituitary–adrenal axis and neuroendocrine systems, prompting the secretion of hormones like catecholamines and arginine vasopressin, which increases BP and HR (30). We wanted to know whether vestibular function training could reduce MS symptoms by decreasing sympathetic nervous system activation. Although the precise mechanism by which vestibular function training reduces sympathetic nervous system activity is unknown, animal and human studies (31) suggest that autonomic nervous system regulation can be altered by vestibular function training. Therefore, we hypothesized that vestibular function training could reduce autonomic disorders and MS symptoms by regulating the excitability of the sympathetic nervous system.

### 4.2 Analysis of Graybiel’s score results

As shown in Figure 1D, the Graybiel score of subjects decreased significantly after 7 days of training, indicating that MS subjective symptoms gradually decreased with the increase of training times, which was consistent with the results of previous studies (24, 32). This pattern may be caused by the establishment of new memory information in the vestibular system and hippocampus, which replaces previous memory information. After repeated exposure to the same stimuli, the body’s response to those stimuli decreases, leading to a reduction in autonomic responses and MS symptoms. Thus, our findings further substantiate the neural mismatch theory, providing theoretical support for treating patients with MS.

### 4.3 Analysis of heart rate variability, heart rate, and blood pressure results

Compared with before training, systolic and diastolic blood pressure were significantly decreased (Figures 1A,B), and LF/HF was significantly increased after training with an electric rotating chair and visual-motion cage rotating chair; In addition, LF, HF, pNN50, and rMSSD were also significantly reduced after training with the visual-motion cage rotating chair. HF reflects the activity of the cardiac vagus nerve, LF reflects the activity of the sympathetic nervous system, the LF/HF ratio reflects the balance between sympathetic and vagal nerve activity, and rMSSD and pNN50 reflect sudden changes in RR intervals, indicating vagal nerve activity (17, 33). Consistent with prior research, we found that both electric rotating chair and visual-motion cage rotating chair training could increase vagal tone and alleviate MS symptoms (34–37). However, Wang et al. (4) found no significant changes in BP after three-dimensional roller training, which is inconsistent with our findings. This discrepancy might be related to the different vestibular organs affected by different training equipment: the electric rotating chair and visual-motion cage rotating chair used in our study only stimulate autonomic responses which are driven by the vestibular semicircular canal system, but Wang et al. used a three-dimensional roller which stimulated both the semicircular canals and the otolith system and thus more comprehensively affected vestibular autonomic responses. It is clear from our study, however, that both electric rotating chairs and visual-motion cage rotating chairs can regulate autonomic nervous activity. In addition, as shown in Figure 1C, there was no significant difference in the heart rate of the subjects in this study before and after the training, which was also contradictory to the experimental results of Wang Xiangxu. This may be affected by the combined effect of the vestibular-sympathetic reflex regulation and the negative feedback regulation of the carotid sinus and aortic arch baroreceptors.

### 4.4 The training results of the two groups were compared and analyzed

In terms of improving the subjective symptoms of motion sickness, the electric swivel chair group was better than the visual cage swivel chair group, which may be related to the following factors: (1) Conflict between visual and vestibular information: In visual-motion cage rotating chair group, the direction of movement perceived by the visual system (counterclockwise rotation generated by the black and white checkboard) and the direction of movement perceived by the vestibular system (clockwise rotation generated by the electric swivel chair) is inconsistent, and this sensory conflict may lead to difficulties in integrating this information in the brain, thus affecting the improvement effect of motion sickness. The electric rotating chair group avoided this sensory conflict, allowing the vestibular system to adapt to stimuli more efficiently. (2) The specificity of acclimatization training: Acclimatization training through repeated exposure to a particular stimulus to the vestibular system gradually and to reduce the motion sickness reaction process. Through continuous and stable vestibular stimulation, the electric rotating chair group makes the acclimation training more specific and helps the vestibular system to form a more stable and lasting adaptability. However, the compound stimulation of the visual-motion cage rotating chair group may make the acclimation effect complicated and unstable, affecting the final improvement effect. (3) Central nervous system integration and regulation: In the process of acclimation training, the central nervous system needs to integrate information from different sensory organs and regulate the body’s response. When vision conflicts with vestibular information, the excitability of vestibular organs is further increased. This excessive excitability may make the vestibular system more sensitive and strong in response to motor stimuli, and the central nervous system may need more time to process and regulate this information, thus affecting the improvement effect of motion sickness. (4) Sensitivity of the vestibular system to rotational stimulation: The vestibular system is particularly sensitive to rotational motion, and the electric rotating chair stimulates the vestibular system through rotational motion. Therefore, the electric rotating chair group may stimulate and train this property of the vestibular system more directly, thus improving the symptoms of motion sickness more effectively. Although the visual-motion cage rotating chair training also provides visual and vestibular rotation stimulation, there may be differences between this stimulation and the actual perception of the vestibular system, resulting in less effective training than the electric rotating chair group. (5) The influence of psychological factors: When subjects receive training, psychological factors may also have an impact on the training effect. Due to the specificity and stability of its training, the electric rotating chair group may be more helpful for patients to build confidence and reduce fear and anxiety about training, thereby improving the training effect. At the same time, we found that there was almost a statistical difference in MSSQ between the two groups, which may be because MSSQ-S scores are related to personal experience, and some of the subjects who filled out the questionnaire had no experience of flying, sailing, or playing in an amusement park, which would make them score lower, but it does not mean that they were free of motion sickness. The MSSQ-S questionnaire has only the screening and qualitative significance of motion sickness, and the “gold standard” of motion sickness is still the score of the Graybiel score.

### 4.5 Analysis of subgroup results

The results of our subgroup analysis showed that, in the low susceptibility group, the electric rotating chair group had lower Graybiel scores than the visual-motion cage rotating chair group. However, the visual-motion cage rotating chair was more effective at improving LF, rMSSD, and pNN50 in high-susceptibility individuals. These results could be related to the following factors: (1) The multidimensional nature of vestibular stimulation: During the training process, the visual-motion cage rotating chair group not only involves the physical rotation stimulus but also includes the visual stimulus (such as the rotation of the cage and changes in the visual environment). This multi-dimensional vestibular stimulation activates the vestibular system more fully, which triggers a stronger autonomic response. (2) Interaction between the vestibule and visual system: During training, there was a closer interaction between the vestibule system and the visual system. This interaction not only enhances the effect of vestibular stimulation but may also promote the vestibular system’s regulation of the autonomic nervous system. As a result, the visual-motion cage rotating chair group showed a more pronounced autonomic response after training, such as reduced LF, rMSSD, and pNN50. In contrast, the electric rotating chair group mainly provides physical rotational stimulation and may not fully activate the vestibular system. Therefore, compared with the electric rotating chair, the visual-motion cage rotating chair stimulated the vestibular system of the study subjects more. In addition, compared with those with low susceptibility, the vestibular organs of those with high susceptibility are more sensitive, and the function of the autonomic nervous system is more likely to be disturbed, so the training can better regulate the sympathetic nerve and parasympathetic nerve function of those with high susceptibility. (3) The excitability of vestibular organs of the two subgroups of study subjects is different: the activity of the body will stimulate the semicircular canal when the stimulation is too large, the body will produce nausea, vomiting, pale and other vestibular autonomic nervous system symptoms, and the degree of these reactions is related to the excitability of vestibular organs. Those with low susceptibility had lower sensitivity to vestibular organs and fewer symptoms such as nausea and vomiting after training. This would explain why the electric rotating chair group had better Graybiel scores than the visual-motion cage rotating chair group in the low susceptibility subgroup.

### 4.6 Advantages

This study provides a roadmap for MS treatment. Specifically, our findings suggest that future treatments should be tailored according to MS susceptibility. Low-susceptibility individuals could be treated with electric rotating chairs, while high-susceptibility individuals could benefit from visual- motion cage rotating chairs. Both options, however, are convenient, cost-effective rehabilitation therapies for MS patients. In addition, the electric swivel chair training and visual cage swivel chair training in this study, as an effective treatment for motion sickness in modern times, have a wide range of application value in real life. These training methods can not only help patients overcome the symptoms of motion sickness but also provide necessary adaptive training for workers in specific industries. For patients with motion sickness, electric rotating chair training is also an effective rehabilitation method. Under the guidance of doctors, patients can gradually increase the difficulty of training, gradually reduce the symptoms of motion sickness, and improve the quality of life. For pilots, through training in electric rotating chairs and visual-motion cage rotating chairs, they can gradually adapt to motion sickness stimulation in the simulated high-speed rotation and complex flight environment. This can not only improve the motion sickness tolerance of pilots but also help them maintain a stable psychological state in actual flight and improve combat effectiveness. In addition, visual-motion cage rotating chair training has important applications in neuroscience research. This training can stimulate the brain’s visual and vestibular systems, helping researchers understand the function and interaction of the two systems. With this training, researchers can explore the mechanisms of perception, cognition, and motor control of the brain, providing new insights and ideas for the development of neuroscience.

### 4.7 Limitations and prospects

However, this study has the following shortcomings: (1) The population of this study was limited to college students, which poses a challenge to the ability to extrapolate the results. College students are generally young, and their physiological and psychological states may be different from those of other age groups. Therefore, there may be risks in applying the findings directly to other populations, such as children, the elderly, or people in specific occupations. To overcome this deficiency, future studies could expand the sample to include people of different age groups, occupations, and cultural backgrounds to improve the universality and accuracy of the study. (2) In this study, only the subjects who filled out the MSSQ-S questionnaire were considered when selecting the population, and visual motion sickness was not considered. Visual motion sickness is a motion sickness symptom related to visual stimulation, which may have different pathogenesis and clinical manifestations from motion sickness. Therefore, ignoring visual motion sickness may lead to incomplete and inaccurate findings. To more fully understand the pathogenesis and influencing factors of motion sickness, future research should consider both motion sickness and visual motion sickness and explore the association and difference between them. At the same time, long-term follow-up studies can be carried out to observe the duration of the effect of motion sickness. (3) This study is only based on the theory and the consistency of the results and lacks the support of objective indicators such as blood tests and neurophysiological tests. Although the consistency of theory and results can verify the reliability of the study to some extent, the lack of support from objective indicators may limit the power of the results. To enhance the scientific validity and credibility of the research, future research can introduce blood tests, neurophysiological tests, and other methods to obtain more objective and accurate physiological and pathological indicators, and to evaluate the treatment effect of motion sickness more comprehensively. (4) In this study, the subjective and objective indexes of the subjects were measured immediately after the last vestibular function training instead of using the same stimulation protocol after the two groups of training. We considered that the visual-motion cage rotating chair group may become nervous due to unaccustomed eyes closing during electric swiping chair stimulation, thus affecting the results, and we still used the data after the last training as the results. This is one of the limitations of this paper. At present, few studies are comparing the two vestibular function training methods, and we are still exploring a better solution.

## 5 Conclusion

In summary, we found that seven days of training with both the electric rotating chair and the visual- motion cage rotating chair relieved MS symptoms and enhanced sympathetic regulation. In terms of improving the subjective degree of MS, the electric rotating chair was superior to the visual-motion cage rotating chair. Subgroup analysis results showed that low-susceptibility MS patients’ symptoms were better relieved with electric rotating chair training, while high-susceptibility patients’ symptoms were better relieved with visual-motion cage rotating chair training.

## Data Availability

The raw data supporting the conclusions of this article will be made available by the authors, without undue reservation.
