# Supplementary material for: Effects of two kinds of vestibular function training on reducing motion sickness in college students
Source: Front Neurol. 2025 Feb 6;16:1433065. doi: 10.3389/fneur.2025.1433065 (PMC11840874; doi:10.3389/fneur.2025.1433065)
Supplement: Supplementary file 1 [file Table_1.docx]

Supplementary Material

**
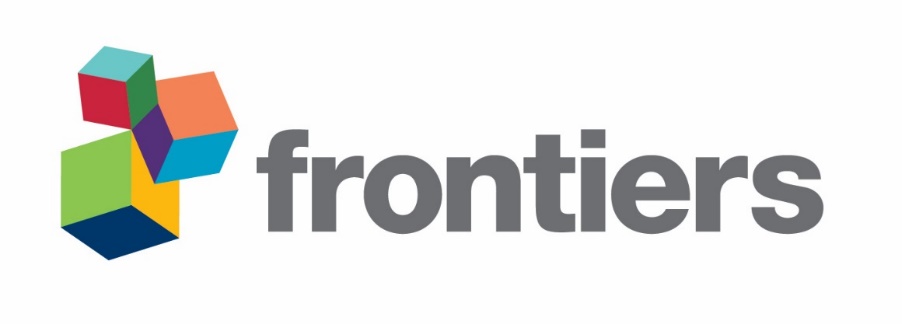
**

**Supplementary Table 1.**

Supplementary Table 1 Graybiel questionnaire

| Signs | Symptom | Score(points) |
| --- | --- | --- |
| Nausea Syndrome | Vomiting or desire to vomit | 16 |
|  | Moderate, severe (marked) nausea | 8 |
|  | Mild nausea | 4 |
|  | Upper abdominal discomfort | 2 |
|  | Sensation in the upper abdomen | 1 |
|  | No unusual sensations. | 0 |
| Skin Color | Severe (marked) pallor | 16 |
|  | Moderate pallor | 8 |
|  | Mild pallor | 4 |
|  | Mild facial flushing | 2 |
|  | Normal color | 0 |
| Sweating | Severe (marked) cold sweat | 8 |
|  | Moderate cold sweat | 4 |
|  | Mild cold sweat | 2 |
|  | No cold sweat | 0 |
| Salivation | Severe (marked) salivation | 8 |
|  | Moderate salivation | 4 |
|  | Mild salivation | 2 |
|  | No salivation | 0 |
| Drowsiness | Severe (apparent) drowsiness | 8 |
|  | Moderate drowsiness | 4 |
|  | Mild drowsiness | 2 |
|  | No drowsiness | 0 |
| Headache | Severe (obvious) headaches | 1 |
|  | Moderate headaches | 1 |
|  | Mild headaches | 1 |
|  | No headaches | 0 |
| Central Nervous Symptom | Dizziness, not relieved by closing eyes | 1 |
|  | Closing the eyes can relieve | 1 |
|  | Dizziness, which can be relieved without closing the eyes | 1 |
|  | No giddiness | 0 |

**Supplementary Table 2.**

Supplementary Table 2 Baseline comparison of low and high susceptibility subjects

| Variable | Low Susceptibility | | *t/Z/χ^2^* | *P* | High Susceptibility | | *t/Z/χ^2^* | *P* |
| --- | --- | --- | --- | --- | --- | --- | --- | --- |
|  | Electric rotating chair group  (*n*=28) | Visual-motion cage rotating chair group  (*n*=27) |  |  | Electric rotating chair group  (*n*=27) | Visual-motion cage rotating chair group  (*n*=27) |  |  |
| Age, year | 19.0  (18.0,21.0) | 20.0  (19.0,22.0) | 0.878^c^ | 0.424 | 20.0  (19.0,20.0) | 20.0  (19.0,21.0) | 0.680^c^ | 0.744 |
| Case, m/f | 28.0  (10.0/18.0) | 27.0  (7.0/20.0) | 0.617^a^ | 0.432 | 27.0  (6.0/21.0) | 27.0  (8.0/19.0) | 0.386^a^ | 0.535 |
| MSSQ-S, points | 6.6  (3.7,36.9) | 6.4  (3.9,12.8) | 0.917^c^ | 0.370 | 41.6  (36.0,45.0) | 38.3  (32.4,42.0) | 1.225^c^ | 0.100 |
| Systolic pressure, mmHg | 128.5  (118.3,138.0) | 121.0  (107.0,131.0) | -1.735 ^c^ | 0.083 | 131.63±17.87 | 125.56±12.79 | 1.436^b^ | 0.157 |
| Diastolic pressure, mmHg | 85.5  (78.0,89.8) | 80.0  (73.0,83.0) | -1.853^c^ | 0.064 | 83.89±13.28 | 80.30±8.52 | 1.184^b^ | 0.242 |
| Heart rate, rpm | 82.82±14.81 | 81.37±11.16 | 0.409^b^ | 0.684 | 77.0  (72.0,89.0) | 80.0  (74.0,84.0) | -0.312^c^ | 0.755 |
| LF, (ms²/Hz) | 1343.3  (909.0,1676.5) | 1228.3  (776.5,1819.6) | -0.185^c^ | 0.853 | 1189.4  (921.9,1617.5) | 1537.9  (986.9,1801.3) | -1.739^c^ | 0.082 |
| HF, (ms²/Hz) | 696.3  (372.1,1100.5) | 586.37  (365.53,940.59) | -0.556^c^ | 0.578 | 761.3  (430.6,1181.0) | 701.9  (565.0,1207.9) | -0.355^c^ | 0.723 |
| LF/HF | 2.0  (1.3,2.6) | 2.21  (1.40,3.02) | -0.682^c^ | 0.495 | 1.7  (1.3,2.8) | 2.1  (1.5,2.6) | -1.159^c^ | 0.246 |
| rMSSD, ms | 37.8  (27.3,48.8) | 34.19  (29.03,43.86) | -0.244^c^ | 0.807 | 38.5  (28.6,52.5) | 39.8  (37.2,48.0) | -0.476^c^ | 0.634 |
| pNN50, % | 15.8  (7.9,25.8) | 14.08  (8.17,25.34) | -0.017^c^ | 0.987 | 20.09±14.53 | 21.01±9.86 | -0.274^d^ | 0.786 |
| Graybiel, points | 4.0  (2.0,5.0) | 3.0  (1.0,6.0) | -0.657^c^ | 0.511 | 13.0  (10.0,18.3) | 16.0  (12.0,24.0) | -1.647^c^ | 0.100 |

^a^: Chi-square tests; ^b^: Two independent sample *t*-tests; ^c^: Kolmogorov-Smirnov *Z* tests; ^d^: Satterthwaite *t*-tests.
